# Identification of Gain and Loss of Function Missense Variants in MRGPRX2’s Transmembrane and Intracellular Domains for Mast Cell Activation by Substance P

**DOI:** 10.3390/ijms20215247

**Published:** 2019-10-23

**Authors:** Chalatip Chompunud Na Ayudhya, Saptarshi Roy, Ibrahim Alkanfari, Anirban Ganguly, Hydar Ali

**Affiliations:** Department of Basic and Translational Sciences, University of Pennsylvania, School of Dental Medicine, Philadelphia PA-19104, USA; chalatip@upenn.edu (C.C.N.A.); roysapta@upenn.edu (S.R.); ibrahim.alkanfari@gmail.com (I.A.); anirban711@aol.com (A.G.)

**Keywords:** mast cells, MRGPRX2, missense variants, substance P, neurogenic inflammation

## Abstract

The neuropeptide substance P (SP) contributes to neurogenic inflammation through the activation of human mast cells via Mas-related G protein-coupled receptor-X2 (MRGPRX2). Using pertussis toxins and YM-254890, we demonstrated that SP induces Ca^2+^ mobilization and degranulation via both the Gαi and Gαq family of G proteins in rat basophilic leukemia (RBL-2H3) cells stably expressing MRGPRX2. To determine the roles of MRGPRX2’s transmembrane (TM) and intracellular domains on SP-induced responses, we utilized information obtained from both structural modeling and naturally occurring MRGPRX2 missense variants. We found that highly conserved residues in TM6 (I225) and TM7 (Y279) of MRGPRX2 are essential for SP-induced Ca^2+^ mobilization and degranulation in transiently transfected RBL-2H3 cells. Cells expressing missense variants in the receptor’s conserved residues (V123F and V282M) as well as intracellular loops (R138C and R141C) failed to respond to SP. By contrast, replacement of all five Ser/Thr residues with Ala and missense variants (S325L and L329Q) in MRGPRX2’s carboxyl-terminus resulted in enhanced mast cell activation by SP when compared to the wild-type receptor. These findings suggest that MRGPRX2 utilizes conserved residues in its TM domains and intracellular loops for coupling to G proteins and likely undergoes desensitization via phosphorylation at Ser/Thr residues in its carboxyl-terminus. Furthermore, identification of gain and loss of function MRGPRX2 variants has important clinical implications for SP-mediated neurogenic inflammation and other chronic inflammatory diseases.

## 1. Introduction

Mast cells (MCs) are tissue-resident granulocytes of hematopoietic origin that play a pivotal role in the inflammatory processes due to their ability to release a wide array of proinflammatory mediators and recruit various immune cells upon stimulation [1,2,3]. MCs are widely distributed throughout the body and are found in close proximity to peripheral nerve endings in various tissues including skin, gastrointestinal mucosa, and respiratory tract [4]. In addition to close anatomic localization, accumulating evidence suggests bidirectional functional communication between MCs and neurons, providing a significant link between the immune and nervous systems [4,5]. MC-derived mediators such as histamine and tryptase activate receptors on sensory nerve endings, resulting in the release of neuropeptides including substance P (SP) which, in turn, evokes further MC activation [5,6,7,8]. Activation of MCs by SP leads to their degranulation, resulting in vasodilation, plasma extravasation, and the recruitment of immune cells including lymphocytes, neutrophils, and macrophages [5,9,10]. Immune cell recruitment further amplifies local inflammatory responses and facilitates peripheral nerve sensitization, which are critical characteristics of neurogenic inflammation [10]. SP-induced MC activation has been implicated in the pathogenesis of pain and many chronic inflammatory diseases such as sickle cell disease [11], atopic dermatitis [12], and chronic idiopathic urticaria [13].

The biological effects of SP were previously thought to be mediated via its canonical neurokinin-1 receptor (NK-1R) [9,14,15]. Several antagonists of this receptor have been developed as potential therapies for a variety of conditions including chemotherapy-induced nausea, inflammation, and pain. While NK-1R antagonists are effective in the treatment of chemotherapy-induced nausea and vomiting, they fail to demonstrate significant anti-inflammatory and analgesic effects [14,15]. This raises the interesting possibility that the nociceptive and proinflammatory actions of SP may be mediated via alternative mechanisms. Recent studies have demonstrated that SP activates human and murine MCs via Mas-related G protein-coupled receptor-X2 (MRGPRX2) and Mrgprb2, respectively [16,17]. Expression of MRGPRX2 is upregulated in human skin MCs of patients with chronic idiopathic urticaria when compared to healthy individuals [13]. A recent study by Serhan et al. [12] demonstrated that SP released from sensory neurons activates murine skin MCs via Mrgprb2 and contributes to the development of atopic dermatitis. Furthermore, Green et al. [18] showed that inflammatory and thermal hyperalgesia requires Mrgprb2-mediated recruitment of immune cells at the injury site. They also demonstrated that SP promotes the release of multiple pro-inflammatory cytokines and chemokines from human MCs via activation of MRGPRX2 [18]. Taken together, these findings suggest that MRGPRX2/Mrgprb2 participate in neurogenic inflammation, chronic urticaria, atopic dermatitis, and pain [12,13,18,19]. However, the molecular mechanism by which MRGPRX2 is activated in response to SP has not been determined.

All G protein-coupled receptors (GPCRs) are structurally similar containing seven transmembrane (TM) α-helices. Binding of ligands to the receptor from the extracellular site promotes the opening of TM6, which results in conformational changes in the cytoplasmic side of membrane, leading to allosteric activation of G proteins [20,21,22]. Venkatakrishnan et al. [22] analyzed the pattern of contact between structurally equivalent residues from the crystal structures of 27 class A GPCRs. From this analysis, it became clear that, upon receptor activation, there is a highly conserved reorganization of residue contacts in TM3 (3x46), TM6 (6x37), and TM7 (7x53) [22]. In this GPCR numbering scheme, the first number denotes the TM domains (1–7) and the second number indicates the residue position relative to the most conserved position, which is assigned the number 50 [23,24]. Thus, 3x46 denotes a residue in TM3, which is at four positions before the most conserved residue (3x50). Similarly, 7x53 denotes a residue in TM7, which is at three positions after the most conserved residue (7x50). Mutations of residues 3x46, 6x37, and 7x53 in a number of class A GPCRs result in significant reduction of G protein activation and downstream signaling, confirming the roles of these positions for the activation of different G proteins [22,25]. In addition to TM domains, conserved residues present in the second intracellular loop (ICL2) of a number of class A GPCRs are involved in coupling to G proteins [26,27]. MRGPRX2 is a member of the class A GPCR family, but the possibility that residues 3x46, 6x37, and 7x53 and conserved residues present in its ICL2 couple to G proteins to cause MC activation has not been tested.

In addition to G proteins, most class A GPCRs signal via another pathway that involves phosphorylation of the receptors at Ser/Thr residues in their carboxyl-terminus by GPCR kinases and the recruitment of adapter proteins known as β-arrestins [28,29,30,31]. This pathway has been implicated in the regulation of GPCR desensitization (uncoupling of the G protein from the receptor), endocytosis, and internalization [30]. GPCR agonists that preferentially activate G proteins are known as G protein-biased and those activate β-arrestin are known as β-arrestin-biased agonists. However, agonists that activate both pathways are known as balanced agonists [32]. Our original studies using host defense peptide LL-37 as a ligand for MRGPRX2 demonstrated that the receptor is resistant to agonist-induced phosphorylation and desensitization, indicating that it acts as a G protein-biased agonist for the receptor [33]. However, our more recent studies demonstrated that distinct ligands act as balanced or G protein-biased agonists for MRGPRX2 [32]. The carboxyl terminus of MRGPRX2 contains five Ser/Thr residues. However, the possibility that these potential phosphorylation sites contribute to receptor regulation by SP has not been determined.

Molecular modeling and mutagenesis studies led to the identification of a ligand binding pocket for a number of MRGPRX2 agonists [34,35,36]. We recently demonstrated that naturally occurring missense variants in MRGPRX2’s predicted ligand binding pocket result in loss of function phenotype of MC activation in response to a diverse group of ligands including the neuropeptide SP [36]. The goal of the present study was to utilize both structural information derived crystal structures of other GPCRs and naturally occurring MRGPRX2 missense variants to determine the roles of MRGPRX2’s TM and intracellular (IC) domains on MC activation by SP. The data presented herein identify a number of gain and loss of function of missense variants of MRGPRX2. These findings have important clinical implications with regard to resistance and susceptibility for developing MC-mediated neurogenic inflammation, pain, atopic dermatitis, and chronic urticaria [12,13,18,19].

## 2. Results

### 2.1. MRGPRX2 Mediates SP-Induced MC Activation via Both Gαi and Gαq

In addition to SP, amphipathic peptides such as the cathelicidin LL-37 and human β-defensin-3 activate human MCs via MRGPRX2 [33,37]. We previously showed that while degranulation in response to these agonists is blocked by pertussis toxin (PTx), Ca^2+^ mobilization is not [33,37]. These findings suggest that MRGPRX2 may couple to both PTx-sensitive (Gαi) and insensitive (Gαq) G proteins. To determine the G protein specificity for SP-induced MRGPRX2-mediated responses, we utilized a pharmacological approach using a Gαi-specific inhibitor (PTx) and a Gαq-specific inhibitor (YM-254890) [38]. Rat basophilic leukemia (RBL-2H3), a commonly used model for MC activation, does not endogenously express MRGPRX2. We therefore utilized RBL-2H3 cells stably expressing MRGPRX2 (RBL-MRGPRX2) to determine the effects of SP on MC activation [33,37,39].

**Figure 1:**
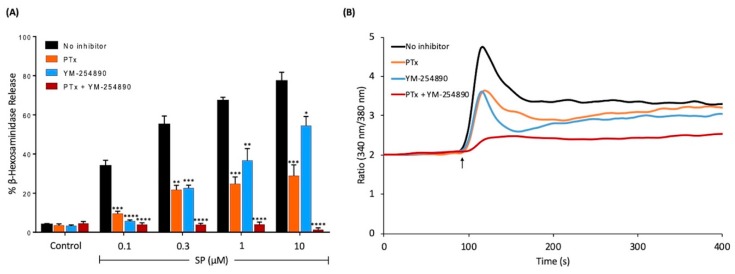
Effects of pertussis toxin (PTx) and YM-254890 on substance P (SP)-induced degranulation and Ca^2+^ mobilization in RBL-2H3 cells stably expressing MRGPRX2, (RBL-MRGPRX2). (**A**) Cells were cultured overnight in the absence or presence of PTx (100 ng/mL, 16 h), washed and incubated with or without YM-254890 (10 μM) for 5 min. Cells were then exposed to a buffer (control) or different concentrations of SP for 30 min, and β-hexosaminidase release was determined. All data points are the mean ± SEM of at least three experiments performed in triplicate. (**B**) Cells were cultured overnight in the absence or presence of PTx (100 ng/mL, 16 h), then loaded with Fura-2 and intracellular Ca^2+^ mobilizations in response to SP (1 μM) were determined. To determine the effect of Gαq, cells were incubated with YM-254890 (10 μM) for 5 min before stimulating with SP. Data shown are representative of three independent experiments. Statistical significance was determined by the nonparametric *t*-test and two-way ANOVA. * *p* ≤ 0.05, ** *p* ≤ 0.01, *** *p* ≤ 0.001, and **** *p* ≤ 0.0001.

SP has been shown to induce MRGPRX2-mediated MC degranulation in a dose-dependent manner [16]. We found that at a low concentration of SP (0.1 μM), PTx caused substantial inhibition of MC degranulation. However, at higher concentrations of SP, only about 50% of MC degranulation was inhibited by PTx (Figure 1A). A similar inhibitory profile was also observed for the Gαq inhibitor, YM-254890, but the extent of inhibition was lower at high concentrations of SP (1 and 10 μM) (Figure 1A). However, SP-induced degranulation was abolished in cells treated with both PTx and YM-254890 (Figure 1A). We also tested the effects of PTx and YM-254890 alone and in combination on SP-induced Ca^2+^ mobilization. Similar to degranulation, we found that PTx or YM-254890 caused partial inhibition of the SP response but a combination of both inhibitors resulted in almost complete inhibition of SP-induced Ca^2+^ response (Figure 1B). Taken together, these findings suggest that MRGPRX2 utilizes both the Gαi and Gαq families of G proteins for SP-induced MC degranulation.

### 2.2. Mutations of the Highly Conserved Residues 3x46, 6x37, and 7x53 in MRGPRX2 Lead to a Significant Reduction in SP-Induced MC Activation

Based on structural and computational studies, it was proposed that positions 3x46, 6x37, and 7x53 are conserved among class A GPCRs and likely participate in G protein coupling [22]. Amino acids at these positions in MRGPRX2 were identified from the GPCR database (GPCRdb) [24]. Residues at positions 3x46, 6x37, and 7x53 in MRGPRX2 are Val, Ile, and Tyr, respectively. Notably, these residues are either large hydrophobic or aromatic residues which are likely to fulfill the van der Waals criterion and facilitate contact formation during the receptor conformational rearrangement [22].

**Figure 2:**
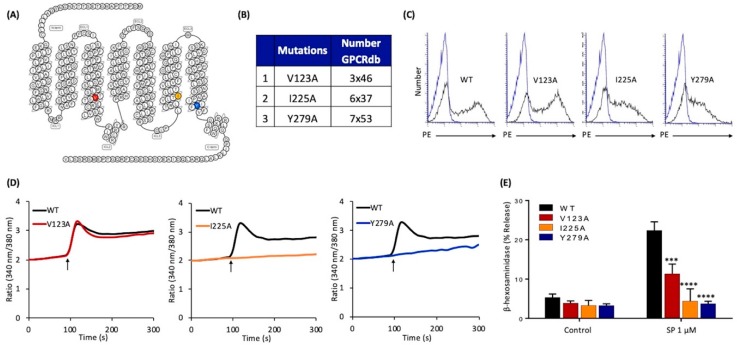
Effects of mutations at MRGPRX2’s highly conserved positions within transmembrane domains (V123A, I225A, and Y279A) on cell surface expression, SP-induced Ca^2+^ mobilization, and degranulation in transiently transfected RBL-2H3 cells. (**A**) Snake diagram of secondary structure of MRGPRX2. Each circle represents amino acid residue with one letter code. Solid red, yellow, and blue backgrounds denote the residues at positions 3x46 (V123), 6x37 (I225), and 7x53 (Y279), respectively; (**B**) amino acid change for each MRGPRX2 mutant.; (**C**) RBL-2H3 cells transiently expressing wild-type (WT)-MRGPRX2 and its mutants were incubated with phycoerythrin (PE)-anti-MRGPRX2 antibody and cell surface receptor expression was determined by flow cytometry. Representative histograms for WT/mutant (black line) and control untransfected cells (blue line) are shown; (**D**) cells expressing WT-MRGPRX2 and its mutants were loaded with Fura-2 and intracellular Ca^2+^ mobilization in response to SP (1 μM) was determined. Data shown are representative of three independent experiments; (**E**) cells were exposed to a buffer (control) or SP (1 μM) for 30 min, and β-hexosaminidase release was determined. All data points are the mean ± SEM of at least three experiments performed in triplicate. Statistical significance was determined by a nonparametric *t*-test. *** *p* ≤ 0.001 and **** *p* ≤ 0.0001.

To determine if these residues in MRGPRX2 contribute to SP-induced MC activation, we first constructed single Ala substitution mutations at these positions, namely V123A, I225A, and Y279A, respectively (Figure 2A,B). We then generated transient transfectants in RBL-2H3 cells. Flow cytometry analysis using phycoerythrin (PE)-conjugated anti-MRGPRX2 antibody showed that these point mutations did not adversely affection cell surface receptor expression (Figure 2C). Interestingly, cells expressing V123A mutant responded normally to SP for Ca^2+^ mobilization but degranulation was inhibited by ~50% when compared to the wild-type (WT) receptor (Figure 2D,E). Although the mutants I225A and Y279A expressed normally on the cell surface (Figure 2C), they did not respond to SP for Ca^2+^ mobilization or degranulation (Figure 2D,E).

### 2.3. Naturally Occurring Missense MRGPRX2 Variants at or Near the Conserved Residues, V123F and V282M, Display Loss of Function Phenotype for SP-Induced MC Activation

**Figure 3:**
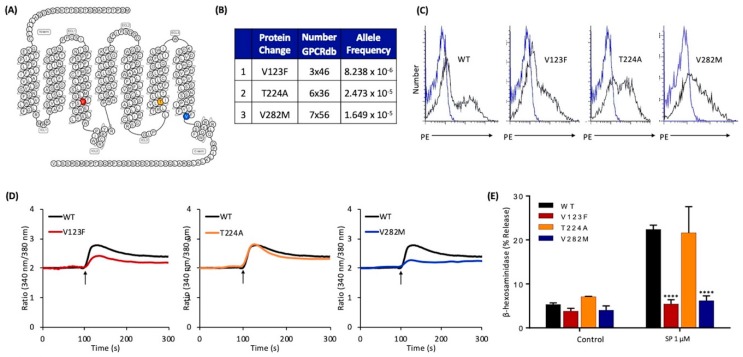
Effects of naturally occurring MRGPRX2 variants at the receptor’s conserved transmembrane domains (V123F, T224A, and V282M) on SP-induced responses in transiently transfected RBL-2H3 cells. (**A**) Snake diagram of secondary structure of MRGPRX2. Each circle represents amino acid residue with one letter code. Solid red, yellow, and blue backgrounds denote the naturally occurring MRGPRX2 variants V123F, T224A, and V282M, respectively; (**B**) amino acid change for each MRGPRX2 variant with allele frequency; (**C**) cell surface expression of WT-MRGPRX2 and its variants was determined by flow cytometry using PE-anti-MRGPRX2 antibody. Representative histograms for WT/variant (black line) and control untransfected cells (blue line) are shown; (**D**) cells expressing WT-MRGPRX2 and its variants were loaded with Fura-2 and intracellular Ca^2+^ mobilization in response to SP (1 μM) was determined. Data shown are representative of three independent experiments; (**E**) cells were exposed to a buffer (control) or SP (1 μM) for 30 min, and β-hexosaminidase release was determined. All data points are the mean ± SEM of at least three experiments performed in triplicate. Statistical significance was determined by nonparametric *t* test. **** *p* ≤ 0.0001.

Next, we searched the GPCRdb [24] to determine if there were any missense MRGPRX2 variants present in the human population with mutations at or near position 3x46, 6x36, or 7x53. We identified three MRGPRX2 variants, namely V123F (3x46), T224A (6x36), and V282M (7x56) (Figure 3A,B). Allele frequency for each variant is shown in Figure 3B. We used the site-directed mutagenesis approach to generate cDNAs encoding each of these variants, which were then transiently transfected in RBL-2H3 cells. Flow cytometry analysis demonstrated that MRGPRX2 and all its variants were expressed on the cell surface (Figure 3C). SP-induced Ca^2+^ mobilization was partially reduced in cells expressing the variant V123F when compared to the WT receptor, but degranulation was completely inhibited (Figure 3D,E). However, cells expressing the variant T224A responded normally to SP for Ca^2+^ mobilization and degranulation (Figure 3B,D,E). By contrast, V282M variant was resistant to both SP-induced Ca^2+^ mobilization and degranulation (Figure 3B,D,E).

### 2.4. Naturally Occurring Missense MRGPRX2 Variants at the Second Intracellular Loop, R138C and R141C, Display Loss of Function Phenotype for SP-Induced MC Activation

**Figure 4:**
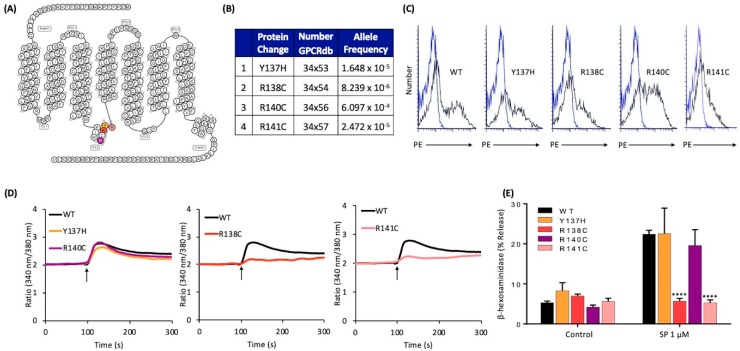
Effects of naturally occurring MRGPRX2 variants at the receptor’s intracellular loops (Y137H, R138C, R140C, and R141C) on SP-induced responses in transiently transfected RBL-2H3 cells. (**A**) Snake diagram of secondary structure of MRGPRX2. Each circle represents amino acid residue with one letter code. Solid yellow, red, purple, and pink backgrounds denote the naturally occurring MRGPRX2 variants; (**B**) amino acid change for each MRGPRX2 variant with allele frequency; (**C**) cell surface expression of WT-MRGPRX2 and its variants was determined by flow cytometry using PE-anti MRGPRX2 antibody. Representative histograms for WT/variant (black line) and control untransfected cells (blue line) are shown; (**D**) cells expressing WT-MRGPRX2 and its variants were loaded with Fura-2 and intracellular Ca^2+^ mobilization in response to SP (1 μM) was determined. Data shown are representative of three independent experiments; (**E**) cells were exposed to a buffer (control) or SP (1 μM) for 30 min, and β-hexosaminidase release was determined. All data points are the mean ± SEM of at least three experiments performed in triplicate. Statistical significance was determined by a nonparametric *t*-test. **** *p* ≤ 0.0001.

Apart from conformational changes in TM helices, recent crystallography and spectroscopy studies on GPCR-heterotrimeric G protein complexes have shown that intracellular loops of the receptors also interact with G proteins and are important for G protein activation [27,40]. Thus, we further searched for naturally occurring missense MRGPRX2 variants in the receptor’s intracellular loops and were able to identify four missense variants within ICL2 (Figure 4A,B). cDNAs encoding these variants were generated and transiently transfected in RBL-2H3 cells. Flow cytometry analysis demonstrated that all four variants expressed on the cell surface (Figure 4C). We found that Y137H and R140C variants responded to SP for Ca^2+^ mobilization and degranulation similar to the WT receptor (Figure 4D,E). By contrast, SP failed to activate these responses in cells expressing R138C and R141C variants (Figure 4D,E).

### 2.5. Mutations in Potential Phosphorylation Sites of MRGPRX2 Leads to Enhanced MC Activation in Response to SP

**Figure 5:**
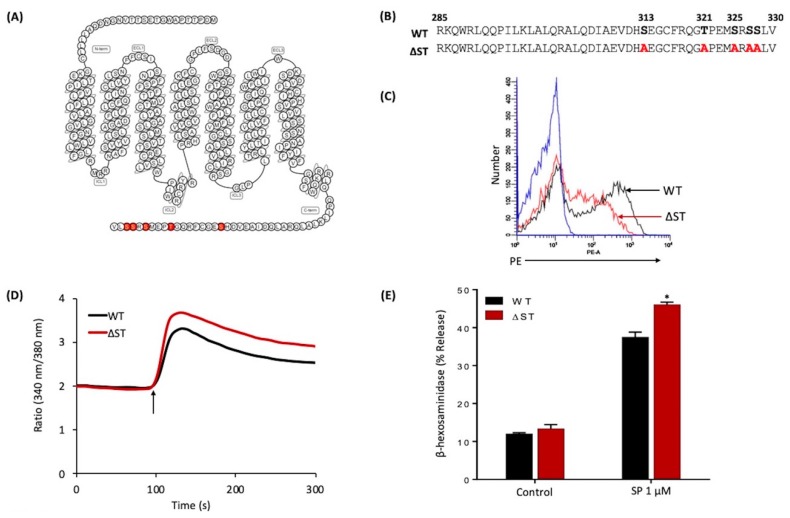
Effects of Ser/Thr residues on MRGPRX2’s carboxyl-terminus on cell surface expression, SP-induced Ca^2+^ mobilization, and degranulation in transiently transfected RBL-2H3 cells. (**A**) Snake diagram of secondary structure of MRGPRX2. Each circle represents amino acid residue with one letter code. Solid red backgrounds denote Ser/Thr residues; (**B**) schematic representation of the carboxyl-terminus of MRGPRX2 (WT) and a phosphorylation-deficient mutant in which all Ser/Thr were replaced with Ala (△ST-MRGPRX2); (**C**) cell surface expression of WT and △ST-MRGPRX2 was determined by flow cytometry using PE-anti-MRGPRX2 antibody. Representative histograms for WT-MRGPRX2 (black line), △ST-MRGPRX2 (red line), and control untransfected cells (blue line) are shown; (**D**) cells expressing WT and △ST-MRGPRX2 were loaded with Fura-2 and intracellular Ca^2+^ mobilization in response to SP (1 μM) was determined. Data shown are representative of three independent experiments; (**E**) cells were exposed to a buffer (control) or SP (1 μM) for 30 min, and β-hexosaminidase release was determined. All data points are the mean ± SEM of at least three experiments performed in triplicate. Statistical significance was determined by a nonparametric *t*-test. * *p* ≤ 0.05.

Phosphorylation of GPCRs by GPCR kinases provides an important mechanism for their desensitization [28,29,31]. Human MRGPRX2 possesses five potential phosphorylation sites at its carboxyl-terminus. To determine the role of MRGPRX2 phosphorylation on SP-induced responses, we generated cDNAs encoding an MRGPRX2 mutant in which all Ser/Thr residues were replaced with alanine (△ST-MRGPRX2) (Figure 5A,B). Transiently transfected RBL-2H3 cells demonstrated reduced cell surface expression of △ST-MRGPRX2, when compared to the WT receptor (Figure 5C). Despite this, SP induced greater Ca^2+^ mobilization and degranulation in cells expressing △ST-MRGPRX2 when compared to the WT receptor (Figure 5D,E).

### 2.6. Naturally Occurring Missense MRGPRX2 Variants at its Carboxyl-Terminus, S325L and L329Q, Display Gain of Function Phenotype for SP-Induced MC Activation

**Figure 6:**
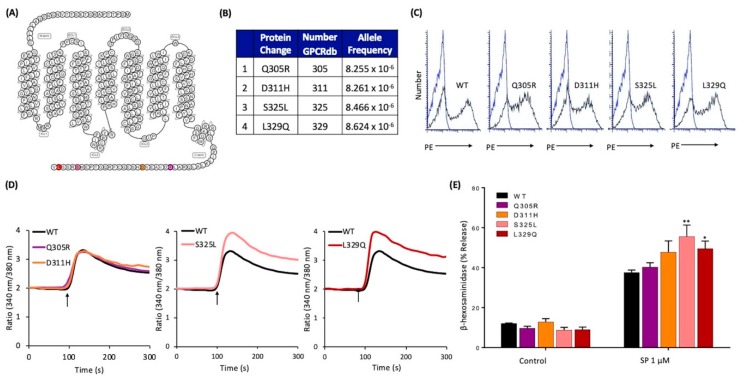
Effects of naturally occurring MRGPRX2 variants within the receptor’s carboxyl-terminus (Q305R, D311H, S325L, and L329Q) on SP-induced responses in transiently transfected RBL-2H3 cells. (**A**) Snake diagram of secondary structure of MRGPRX2. Each circle represents amino acid residue with one letter code. Solid purple, orange, pink, and red backgrounds denote the naturally occurring missense variants Q305R, D311H, S325L, and L329Q, respectively; (**B**) amino acid change for each MRGPRX2 variant with allele frequency; (**C**) cell surface expression of WT-MRGPRX2 and its variants was determined by flow cytometry using PE-anti MRGPRX2 antibody. Representative histograms for WT/variant (black line) and control untransfected cells (blue line) are shown; (**D**) cells expressing WT-MRGPRX2 and its variants were loaded with Fura-2 and intracellular Ca^2+^ mobilization in response to SP (1 μM) was determined. Data shown are representative of three independent experiments; (**E**) cells were exposed to buffer (control) or SP (1 μM) for 30 min, and β-hexosaminidase release was determined. All data points are the mean ± SEM of at least three experiments performed in triplicate. Statistical significance was determined by a nonparametric *t*-test. * *p* ≤ 0.05 and ** *p* ≤ 0.01.

Search of the GPCRdb [24] led to the identification of four missense variants in the carboxyl-terminus of MRGPRX2 (Figure 6A,B), of which one variant results in the replacement of Ser with Leu (S325L). Flow cytometry analysis of transfected RBL-2H3 cells demonstrated equivalent cell surface expression of all variants (Figure 6C). Cells expressing Q305R and D311H variants responded similarly to SP for Ca^2+^ mobilization and degranulation when compared to the WT receptor (Figure 6D,E). By contrast, S325L and L329Q variants displayed higher responses to SP for both Ca^2+^ mobilization and degranulation (Figure 6D,E).

## 3. Discussion

Unique features of MRGPRX2 that differentiate it from other class A GPCRs are that it is expressed predominantly in one subtype of MCs and responds to a variety of cationic ligands, including SP [18,41,42,43]. Structure-based computational modeling and site directed mutagenesis approach have been used to show that negatively charged residues Glu164 (E164) in TM4 (4x60) and Asp184 (D184) in TM5 (5x36) are important for binding opioids and SP [34,35,36]. We recently showed that missense variants in the MRGPRX2’s ligand-binding pocket (G165E and D184H) fail to respond to a variety of cationic ligands including SP, human β-defensin-3, and icatibant (bradykinin B2 receptor antagonist) for receptor activation [36]. In the present study, we utilized information derived from the comparison of crystal structures of a number of class A GPCRs, as well as naturally occurring missense variants in MRGPRX2’s predicted G protein coupling domains and potential phosphorylation sites to identify a number of gain and loss of function variants. These findings have important implications for SP/MRGPRX2-mediated conditions such as neurogenic inflammation, pain, atopic dermatitis, and chronic idiopathic urticaria [12,13,18,19].

In the inactive state of class, A GPCRs, the residue at 6x37 is in contact with a conserved hydrophobic residue at position 3x46 [22]. Upon receptor activation, this interaction is rearranged so that residue at 3x46 breaks contact with residue 637 and forms a new contact with a tyrosine residue, Tyr7x53, within the highly conserved NPXXY motif of TM7 [21,22,26]. This rearrangement results in the activation of G proteins. Accordingly, Ala substitution of each of these residues (3x46, 6x37, and 7x53) of the vasopressin V2 receptor results in its uncoupling from Gαs and Gαq [22]. We showed that MRGPRX2 coupled to both Gαi and Gαq families of G proteins for Ca^2+^ mobilization and degranulation in response to SP. Thus, PTx (a Gαi-specific inhibitor) in combination with YM-254890 compound (a Gαq-specific inhibitor) completely inhibited SP-induced MC activation. By contrast, using either PTx or YM-254890 alone was unable to abolish Ca^2+^ and degranulation responses to SP. Of note, many GPCRs have been shown to display distinct intracellular signaling and cellular responses depend on agonist concentrations [44]. It is possible that low-dose SP induces MRGPRX2 to preferentially couple to either Gαi or Gαq, whereas a high concentration of SP mediates MRGPRX2 conformational change to couple to both G proteins. The data presented herein suggest that similar to other class A GPCRs, residues 3x46, 6x37, and 7x53 in MRGPRX2 contribute to coupling to Gαi and Gαq families of G proteins and that naturally occurring missense variants within or near some of these highly conserved residues may contribute to loss of function phenotype for MC activation by SP. One interesting finding of the present study was that while V123A (3x46) mutation resulted in partial inhibition of SP-induced degranulation, the missense variant V123F (3x46) failed to respond to SP for Ca^2+^ mobilization or degranulation. These findings suggest that the presence of a bulky Phe group in the missense variant V123F less effectively breaks the interaction of 3x46 with 6x37 or blocks the formation of new contact Tyr residue at 7x53. Another interesting finding was that while cells expressing I225A mutation (6x37) were resistant to SP-induced Ca^2+^ mobilization and degranulation, a missense mutation T224A responded normally to SP. However, a missense V282M mutation three amino acids way from the Tyr7x53 in the conserved NPXXY motif resulted in complete loss of function phenotype for SP-induced MC degranulation. This finding likely emphasizes the importance of this region of MRGPRX2 for coupling to G proteins.

Additionally, we identified four missense MRGPRX2 variants at the predicted G protein coupling regions within its ICL2. Crystallography and cryogenic electron microscopy studies of GPCR-heterotrimeric G protein complexes have provided evidence that this ICL interacts with Gα subunit to promote GDP dissociation and subsequent GTP binding, resulting in activation of G proteins [27,40]. Mutations of ICL2 in β_2_ adrenergic receptor have been shown to impair G protein coupling [45]. Here, we found that cells expressing R138C and R141C variants in this region displayed loss of function phenotype in response to SP. By contrast, other MRGPRX2 variants (Y137H and R140C) had no effect on SP-induced MC activation. Intriguingly, this region has also been identified as a cholesterol recognition amino acid consensus (CRAC) motif of MRGPRX2. Cholesterol-rich microdomains (lipid rafts) are membrane microdomains enriched in cholesterol and glycerophospholipids that mediate organization and function of many membrane receptors and biomolecules including GPCRs [46]. The orientation and organization of membrane proteins present in the lipid raft allow greater efficiency and specificity of signal transduction by facilitating protein–protein interactions and preventing crosstalk between competing pathways [46]. Given that MRGPRX2 contains the CRAC motif, it is possible that lipid rafts also contribute to MRGPRX2 activation and G protein coupling. Positively charged Arg residue of MRGPRX2 (R138 and R141) might be necessary to interact with negatively-charged hydroxyl group of cholesterol for proper MRGPRX2 functioning. Substitution of this amino acid with neutral amino acid Cys may disrupt the interaction with lipid raft domains, resulting in loss of function phenotype. The interaction between MRGPRX2 and lipid rafts will be the subject of further investigation to delineate the role of lipid rafts in MRGPRX2 signaling.

While GPCR signaling is essential for regulating physiological function of cells, overstimulation can be deleterious and contributes to pathologic conditions. Thus, following their activation, GPCRs undergo desensitization via phosphorylation of Ser/Thr residues at their carboxyl-terminus [26,31]. Binding of β-arrestin to phosphorylated GPCRs has been implicated in receptor desensitization, endocytosis, and internalization [26,30,31]. It also initiates a distinct downstream signaling pathway known as β-arrestin-mediated activation [26,30,31]. Here, we showed that mutation of all possible phosphorylation sites of MRGPRX2 (△ST-MRGPRX2) leads to significantly higher SP-induced MC activation. We further examined the effects of naturally occurring missense MRGPRX2 mutations within the carboxyl-terminus. Of these, we identified one missense variant in which a potential phosphorylation site is mutated, S325L. Interestingly, cells expressing this variant exhibited gain of function phenotype for MC degranulation in response to SP. These findings are consistent with previous studies in β_2_ adrenergic receptor that demonstrated the importance of distal phosphorylation residues for high-affinity β-arrestin binding and receptor desensitization [47]. Distinct GPCR phosphorylation sites have been proposed to be targeted by different GPCR kinases and establish a specific barcode that imparts distinct conformations to the recruited β-arrestin, thus regulating different functional activities, such as desensitization, internalization, and downstream signaling [47]. It is possible that the S325 of MRGPRX2 is responsible for receptor desensitization, thus mutation in this position leads to enhanced SP-induced responses due to impaired desensitization.

In addition, the carboxyl-terminus of MRGPRX2 contains a class I PDZ (PSD-95/Dlg/Zo1) recognition motif S/T-X-φ (where ‘‘φ’’ indicates hydrophobic amino acid and ‘‘X’’ indicates any amino acid). PDZ proteins have been implicated in regulating receptor desensitization, internalization, and signaling for several GPCRs such as β_2_ adrenergic receptor, parathyroid hormone receptor, and opioid receptors [48]. For example, the PDZ protein, Na^+^/H^+^ exchanger regulatory factor 1 (NHERF1) has been shown to regulate type 1 parathyroid hormone receptor signaling by anchoring the receptor to the plasma membrane, thus restricting its desensitization and internalization [49]. Our lab previously demonstrated that these proteins also promote C3a-induced degranulation in human MCs [50]. Given that MRGPRX2 possesses a class I PDZ motif, it is possible that PDZ proteins such as NHERF1 contributes to the regulation of MRGPRX2. It is also possible that missense mutations in the receptor’s PDZ motif may enhance the interaction with PDZ proteins, resulting in gain of function phenotype. Altogether, our findings herein indicate the significance of carboxyl-terminal residues for MRGPRX2 regulation and activation. The MRGPRX2 mutations at its carboxyl-terminus may lead to gain of function phenotype for SP-induced MC degranulation due to impaired receptor desensitization, enhanced interaction with PDZ proteins, or both.

Taken together, the data presented herein have identified mutations in MRGPRX2 at the regions involved in the receptor activation pathway. Missense MRGPRX2 mutations in the G protein-coupling regions in TMs and ICL2 fail to activate MC in response to SP, presumably due to impaired G protein coupling. By contrast, MRGPRX2 variants at the carboxyl-terminus, which is responsible for receptor phosphorylation and desensitization, lead to higher responses for MC activation. Thus, individuals with loss of function MRGPRX2 mutation, V123F, R138C, R141C, or V282M may display resistance to developing neurogenic inflammation and chronic inflammatory diseases. By contrast, individuals who harbor the gain of function variant, S325L or L329Q, may be more susceptible to develop these conditions.

## 4. Materials and Methods

### 4.1. Materials

All cell culture reagents were obtained from Invitrogen (Gaithersburg, MD, USA). Amaxa transfection kit (Kit V) was obtained from Lonza (Gaithersburg, MD, USA). Q5 Site-Directed Mutagenesis Kit was from New England BioLabs (Ipswich, MA). Substance P (SP) was from AnaSpec (Fremont, CA, USA). Pertussis toxin (PTx) was from List Biological Laboratories (Campbell, CA, USA). YM-254890 was from Wako Chemicals (Richmond, VA, USA) and p-nitrophenyl-N-acetyl-β-D-glucosamine (PNAG) was from Sigma-Aldrich (St. Louis, MO, USA). Fura-2 acetoxymethyl ester was from Abcam (Cambridge, MA, USA). PE-conjugated anti-MRGPRX2 antibody was from BioLegend (San Diego, CA, USA). MRGPRX2 plasmid encoding hemagglutinin (HA)-tagged human MRGPRX2 in pReceiver-MO6 vector was obtained from GeneCopoeia (Rockville, MD, USA).

### 4.2. Cell Culture

Rat basophilic leukemia (RBL-2H3) cells were maintained as monolayer cultures in Dulbecco’s modified Eagle’s medium (DMEM) supplemented with 10% fetal bovine serum (FBS), l-glutamine (2 mM), penicillin (100 IU/mL), and streptomycin (100 µg/mL) at 37 °C with 5% CO_2_ [51]. RBL-2H3 cells stably expressing MRGPRX2 (RBL-MRGPRX2) were maintained similarly in the presence of G-418 (1 mg/mL) [39].

### 4.3. Construction of MRGPRX2 Variants

Q5 site-directed mutagenesis kit (New England BioLabs, Ipswich, MA, USA) was used to generate MRGPRX2 variants in HA-tagged plasmid. To confirm the correct nucleotide sequences, each mutant was verified by DNA sequencing prior to transfection. The forward and reverse primers used for each variant are listed below.

V123A: Forward: 5’-CTGAGCACCGCCAGCACCGAG-3’

Reverse: 5’-CATGCTCAGGCCTGCAAG-3’;

V123F: Forward: 5’-GCTGAGCACCTTCAGCACCGA-3’

Reverse: 5’-ATGCTCAGGCCTGCAAGG-3’;

Y137H: Forward: 5’-GCCCATCTGGCATCGCTGCCG-3’

Reverse: 5’-CACAGGACGGACAGGCAG-3’;

R138C: Forward: 5’-CATCTGGTATTGCTGCCGCCG-3’

Reverse: 5’-GGCCACAGGACGGACAGG-3’;

R140C: Forward: 5’-GTATCGCTGCtGCCGCCCCAG-3’

Reverse: 5’-CAGATGGGCCACAGGACGG-3’;

R141C: Forward: 5’-TCGCTGCCGCTGCCCCAGACA-3’

Reverse: 5’-TACCAGATGGGCCACAGGACGG-3’;

T224A: Forward: 5’-GCTGTACCTGGCCATCCTGCT-3’

Reverse: 5’-CTGGTCAGTGGCAGACCC-3;

I225A: Forward: 5’-GTACCTGACCGCCCTGCTCACAGTGC-3’

Reverse: 5’-AGCCTGGTCAGTGGCAGA-3’;

Y279A: Forward: 5’-CCCCATCATTGCCTTCTTCGTGG-3’

Reverse: 5’-TTGGCACTGCTGTTAAGAG-3’;

V282M: Forward: 5’-TTACTTCTTCATGGGCTCTTTTAGG-3’

Reverse: 5’-ATGATGGGGTTGGCACTG-3’;

Q305R: Forward: 5’-AGGGCTCTGCGGGACATTGCT-3’

Reverse: 5’-CTGGAGAGCCAGCTTGAG-3’;

D311H: Forward: 5’-TGCTGAGGTGCATCACAGTGAAG-3’

Reverse: 5’-ATGTCCTGCAGAGCCCTC-3’;

S325L: Forward: 5’-CCGGAGATGTTGAGAAGCAGTCTG-3’

Reverse: 5’-GGTGCCCTGACGGAAGCA-3’;

L329Q: Forward: 5’-AGAAGCAGTCAGGTGTAGCTCGAG-3’

Reverse: 5’-CGACATCTCCGGGGTGCC-3’

MRGPRX2 phosphorylation-deficient mutant (Ser/Thr residues mutated to Ala; △ST-MRGPRX2) was generated by PCR. Construct was verified by DNA sequencing prior to transfection. The forward and reverse primers used are listed below.

△ST-MRGPRX2: Forward: 5’-ACATCCGCGGACCATGTACCCTTACGACGTCCCAGACTAC

GCTGATCCAACCACCCCGGCCTGGGGAACAGAA-3’

Reverse: 5’-ACATCTCGAGCTACACCAGAGCGGCTCTCGCCATCTCCGGGGCGCCCTGAC

GGAAGCATCCTTCAGCGTGATC-3’

### 4.4. Generation of Cells Transiently Expressing MRGPRX2 and its Variants

RBL-2H3 cells transiently expressing MRGPRX2 or its missense variants were generated as described previously [36]. Briefly, cells (2 × 10^6^) were transfected with 2 µg of HA-tagged plasmid using the Amaxa Nucleofector Device and Amaxa Kit V according to the manufacturer’s protocol. Cells were used within 16–20 h after transfection.

To detect cell surface MRGPRX2 and its variants’ expression, transfected RBL-2H3 cells (0.5 × 10^6^) were incubated with PE-conjugated anti-MRGPRX2 antibody for 30 min at 4 °C in the dark, washed in FACS buffer (PBS containing 2% FCS and 0.02% sodium azide), and fixed in 1.5% paraformaldehyde. Cells were acquired using a BD LSR II flow cytometer (San Jose, CA, USA). Results were analyzed using WinList software, version 8.

### 4.5. Degranulation Assay

The degranulation was measured by β-hexosaminidase release as described previously [51]. Briefly, transfected RBL-2H3 cells (5 × 10^4^ cells per well) were seeded into a 96-well, white, clear-bottom cell culture plate and incubated overnight in a 37 °C incubator with 5% CO_2_. To determine the inhibitory effects of PTx and YM-254890 on MC degranulation, cells were pretreated with PTx (100 ng/mL, 16 h) and/or YM-254890 (10 μM, 5 min) prior to stimulation with SP. Cells were then washed twice and suspended in a total volume of 50 μL HEPES buffer containing 0.1% bovine serum albumin (BSA). Experimental groups were stimulated with SP for 30 min at 37 °C. Cells without treatment were designated as controls. To determine the total β-hexosaminidase release, unstimulated cells were lysed in 50 μL of 0.1% Triton X-100. Aliquots (20 μL) of supernatants or cell lysates were incubated with 20 μL of 1 mM p-nitrophenyl-*N*-acetyl-β-*D*-glucosamine (PNAG) for 1 h at 37 °C. The reaction was stopped by adding 250 μL of stop buffer (0.1 M Na_2_CO_3_/0.1 M NaHCO_3_). The β-hexosaminidase release was assessed by measuring absorbance at 405 nm using Versamax microplate spectrophotometer (Molecular Devices, San Jose, CA, USA).

### 4.6. Calcium Mobilization Assay

Transfected RBL-2H3 cells (2 × 10^6^) were loaded with 1 μM Fura-2 acetoxymethyl ester for 30 min at 37 °C, followed by de-esterification in HEPES-buffered saline for additional 15 min at room temperature. Cells were washed, resuspended in 1.5 mL of HEPES-buffered saline containing 0.1% BSA, and then stimulated with SP. In some experiments, cells were treated with PTx (100 ng/mL, 16 h) and/or YM-254890 (10 μM, 5 min), and then stimulated with SP. Ca^2+^ mobilization was determined using a Hitachi F-2700 Fluorescence Spectrophotometer with dual excitation wavelength of 340 and 380 nm, and an emission wavelength of 510 nm.

### 4.7. Statistical Analysis

Data shown are mean ± standard error of the mean (SEM) values derived from at least three independent experiments. GraphPad Prism scientific software version 6.07 was used for statistical analysis. Statistical significance was determined using a nonparametric *t*-test and two-way ANOVA due to non-normal distribution data. Differences were considered as statistically significant at a value of * *p* ≤ 0.05, ** *p* ≤ 0.01, *** *p* ≤ 0.001, and **** *p* ≤ 0.0001.

## Abbreviations

MC	Mast cell
SP	Substance P
NK-1R	Neurokinin-1 receptor
MRGPRX2	Mas-related G protein-coupled receptor-X2
GPCR	G protein-coupled receptor
TM	Transmembrane
ICL	Intracellular loop
PTx	Pertussis toxin
RBL-2H3	Rat basophilic leukemia-2H3 cells
RBL-MRGPRX2	RBL-2H3 cells stably expressing MRGPRX2
GPCRdb	GPCR database
WT	Wild-type
PE	Phycoerythrin
CRAC	Cholesterol recognition amino acid consensus
NHERF	Na^+^/H^+^ exchanger regulatory factor
